# Association Between Long-Term Exposure to Ambient Air Pollution and Fasting Blood Glucose: A Systematic Review and Meta-Analysis

**DOI:** 10.3390/toxics12110792

**Published:** 2024-10-30

**Authors:** Tong Wu, Yang Lan, Ge Li, Kai Wang, Yu You, Jiaqi Zhu, Lihua Ren, Shaowei Wu

**Affiliations:** 1 Department of Occupational and Environmental Health, School of Public Health, Xi’an Jiaotong University Health Science Center, Xi’an 710061, China; 2 Key Laboratory of Environment and Genes Related to Diseases (Xi’an Jiaotong University), Ministry of Education, Xi’an 710061, China; 3 Key Laboratory of Trace Elements and Endemic Diseases in Ministry of Health, Xi’an 710061, China; 4 Key Laboratory for Disease Prevention and Control and Health Promotion of Shaanxi Province, Xi’an 710061, China; 5 Shaanxi Provincial Center for Disease Control and Prevention (Shaanxi Provincial Institute for Endemic Disease Control), Xi’an 710061, China; 6 School of Nursing, Peking University, Beijing 100871, China

**Keywords:** air pollution, fasting blood glucose, gaseous pollutant, meta-analysis, particulate matter

## Abstract

Increasing studies are indicating a potential association between ambient air pollution exposure and fasting blood glucose (FBG), an indicator of prediabetes and diabetes. However, there is inconsistency within the existing literature. The aim of this study was to summarize the associations of exposures to particulate matters (PMs) (with aerodynamic diameters of ≤1 μm (PM_1_), ≤2.5 μm (PM_2.5_), and ≤10 μm (PM_10_), respectively) and gaseous pollutants (sulfur dioxide (SO_2_), nitrogen dioxide (NO_2_) and ozone (O_3_)) with FBG based on the existing epidemiological research for a better understanding of the relationship between air pollution and diabetes. Up to 2 July 2024, we performed a comprehensive literature retrieval from various electronic databases (PubMed, Web of Science, Scopus, and Embase). Random-effect and fixed-effect models were utilized to estimate the pooled percent changes (%) and 95% confidence intervals (CIs). Then, subgroup meta-analyses and meta-regression analyses were applied to recognize the sources of heterogeneity. There were 33 studies eligible for the meta-analysis. The results showed that for each 10 μg/m^3^ increase in long-term exposures to PM_1_, PM_2.5_, PM_10_, and SO_2_, the pooled percent changes in FBG were 2.24% (95% CI: 0.54%, 3.96%), 1.72% (95% CI: 0.93%, 2.25%), 1.19% (95% CI: 0.41%, 1.97%), and 0.52% (95% CI:0.40%, 0.63%), respectively. Long-term exposures to ambient NO_2_ and O_3_ were not related to alterations in FBG. In conclusion, our findings support that long-term exposures to PMs of various aerodynamic diameters and SO_2_ are associated with significantly elevated FBG levels.

## 1. Introduction

Diabetes is a complex metabolic disorder characterized by hyperglycemia due to dysfunctional insulin secretion or action [1,2]. This disease has become one of the most significant contributors to the global burden of disease and premature mortality [3,4,5]. The number of people with diabetes was estimated to be approximately 529 million in 2021, and this figure was projected to increase to 1.31 billion by 2050 [6]. Globally, 79.2 million disability-adjusted life years (DALYs) lost were caused by diabetes in 2021 [6]. Recent projections indicate an approximate 75% rise in diabetes-related mortality rates from 2016 to 2040 [7].

Major ambient air pollutants consist of particulate matters (PMs) of different sizes (e.g., aerodynamic diameter ≤1 μm (PM_1_), ≤2.5 μm (PM_2.5_), and ≤10 μm (PM_10_)) and gaseous pollutants including sulfur dioxide (SO_2_), nitrogen dioxide (NO_2_), and ozone (O_3_) [4,8]. In 2021, air pollution was ranked as the second leading risk factor for early deaths worldwide, and it was estimated to contribute to approximately 8.1 million deaths, accounting for about 12% of total global deaths [9]. There has been increasing evidence in recent years suggesting that air pollution is an emerging risk factor for diabetes [10,11,12]. The underlying mechanisms may include the following. (1) Overproduction of proinflammatory mediators [5,13]: exposure to air pollutants induces elevated levels of proinflammatory mediators, including tumor necrosis factor-α (TNF-α), C-reactive protein (CRP), interleukin-8 (IL-8), and interleukin-6 (IL-6), which in turn lead to the activation of c-Jun N-terminal kinase (JNK) [14,15,16]. Activated JNK inhibits insulin signaling via serine phosphorylation of the substrate proteins of insulin receptor, leading to the development of insulin resistance and consequently an increased risk of elevated glucose [14,15,16]. (2) Development of oxidative stress [5,13]: exposure to air pollutants induces oxidative stress by increasing reactive oxygen species (ROS) levels, leading to mitochondrial destruction and subsequent β-cell dysfunction [5,17]. In addition, animal experiments have shown that oxidative stress can exacerbate insulin resistance by blocking insulin signaling via the activation of nuclear factor-kappa-B (NF-κB) [18]. (3) Endothelial dysfunction [13]: exposure to air pollutants may exacerbate endothelial dysfunction by inducing inflammation and oxidative stress, whereas endothelial dysfunction may impair the action of insulin in skeletal muscle, inducing blood flow to non-nutritive tissues, which can lead to increased blood glucose levels [13,19,20]. (4) Over-activity of the sympathetic nervous system [21]: exposure to air pollutants leads to sympathetic nervous activation and reduced heart rate variability, which in turn leads to reduced insulin sensitivity and ultimately disturbed glucose metabolism [21,22,23].

Fasting blood glucose (FBG), also known as basal glucose, is widely used by clinicians to diagnose diabetes and prediabetes [2]. Impaired fasting glucose (IFG) refers to an elevated fasting glucose level that does not yet meet the diagnostic criteria for diabetes [2]. People with IFG are considered to have prediabetes, which indicates a high risk of developing diabetes in the future [2]. To date, several epidemiological studies have shown that exposure to ambient air pollution has an adverse impact on FBG. For example, Yang et al. (2018) and Feizi et al. (2023) have demonstrated that long-term exposures to PMs (PM_2.5_ and PM_10_), SO_2_, NO_2_, and O_3_ contribute to an increased risk of elevated FBG [8,24]. However, other studies presented inconsistent results. For example, Holliday et al. (2019) and Liu et al. (2022) did not observe any significant associations between long-term exposures to PMs (PM_2.5_ and PM_10_) and elevated FBG [25,26]. Lin et al. (2020) also found no association between NO_2_ and O_3_, and elevated FBG [27]. Only one meta-analysis has been conducted to investigate the association between exposure to air pollution and FBG. However, this meta-analysis only investigated the associations between PM_2.5_ and PM_10_ exposures and FBG, and did not consider the impact of other ambient air pollutants [28].

This study focused on the potential effects of long-term exposure to air pollution on FBG rather than short-term exposure. According to the WHO report at the 68th World Health Assembly, while short-term and long-term exposures to air pollution can both lead to adverse health effects, long-term exposure to air pollution could be more damaging to health, with many adverse health effects occurring at relatively low levels (below WHO-proposed air quality guidelines levels) [29]. Given the paucity of research on the associations of short-term exposures to ambient PM_1_, SO_2_, NO_2_, and O_3_ with FBG compared to that of long-term exposures, and the fact that the associations of short-term exposures to PM_2.5_ and PM_10_ with FBG have been evaluated in a recent meta-analysis [28], we only comprehensively evaluated the associations of long-term exposures to a variety of ambient air pollutants (PM_1_, PM_2.5_, PM_10_, SO_2_, NO_2_, and O_3_) with FBG based on relevant epidemiological studies.

## 2. Methods

### 2.1. Search Strategy

A systematic search for studies in electronic databases (PubMed, Web of Science, Scopus, and Embase) was conducted up to 2 July 2024. Furthermore, the references of all eligible studies that were not found in the search results were also scrutinized. The search strategies were constructed using a combination of keywords related to air pollution, PMs, SO_2_, NO_2_ or O_3_, and FBG simultaneously. The study was conducted strictly according to the Preferred Reporting Items for Systematic Reviews and Meta-Analyses (PRISMA) statement [30]. Table S1 in the Supplementary Materials contains the full list of specific search phrases.

### 2.2. Inclusion and Exclusion Criteria

Studies were considered according to the following criteria: (1) epidemiological studies that examined the association of ambient air pollution exposure with FBG levels; (2) studies that provided effect estimates (percent changes or regression coefficients) with 95% confidence intervals (CIs) for quantitative estimations of the association of FBG with ambient air pollution exposure; (3) cross-sectional or cohort studies; and (4) literature published in English.

Studies would be rejected according to the following criteria: (1) studies about indoor or occupational exposures; (2) standardized quantitative transformation could not be performed; (3) non-epidemiological investigations, such as toxicological studies or animal experiments; (4) conference proceedings, editorials, case reports, meta-analyses, and review articles; (5) studies about other blood glucose indices (e.g., glycated hemoglobin, random blood glucose); (6) studies examining the potential effects of short-term exposure to targeted air pollution; and (7) the corresponding author(s) could not be reached at the time of extracting the data.

### 2.3. Data Extraction

Two reviewers (T.W. and Y.Y.) individually gathered the following data from each included study: first author, publication year, study area, participant type, sample size, study design, air pollution exposure assessment method, exposure period, FBG levels, effect estimates, and related 95% CIs. If there was a dispute, a third investigator (Y.L.) would make the final decision. A fourth investigator (S.W.) undertook a meticulous evaluation of all the recordings, data extraction, and statistical analysis techniques before submission. Two distinct categories of exposure to ambient air pollution were recognized: short-term exposure and long-term exposure. Short-term exposure is defined as exposure that lasts for a few weeks or days, and may result in acute health effects; whereas long-term exposure is defined as exposure that persists for a minimum of six months and may result in chronic health effect s [31,32]. The present study focused on long-term exposure to air pollution. In instances where multiple exposure windows were provided in a single study, the effect estimate from the exposure window with the most significant effect value (i.e., smallest *p*-value) was selected. In instances where multiple models were employed in a single study, the effect estimates from the single-pollutant model that controlled for the greatest number of covariates were extracted. In instances where multiple studies were conducted on the same population, the study that investigated the most types of ambient air pollutants was included. Furthermore, if the included article lacked the quantitative data we needed, we would endeavor to contact the corresponding author(s). If the corresponding author(s) did not respond to our request for quantitative information, the article in question would be excluded.

### 2.4. Quality Assessment

As the included studies were epidemiological studies with different designs, the Effective Public Health Practice Project (EPHPP) quality assessment tool was used to assess the quality of each study based on six basic criteria, including selection bias, study design, control of confounders, use of blinding, data collection methods, and the presence of withdrawals and drop-outs. Each criterion was rated as good, moderate or poor, and an overall rating was given based on these six criteria [33]. All quality assessments were conducted independently by two investigators (T. W. and Y. Y.). This tool is described in detail in Table S2 (Supplementary Materials).

### 2.5. Statistical Analysis

Percent changes and 95% CIs were used to describe the association of FBG with ambient air pollution. We standardized all the percent changes (%) and 95% CIs to obtain the effect values of incremental exposure to ambient air pollutants at 10 μg/m^3^. The formula is as follows: change % (standardized) = change % (original) × Increment (standardized)/increment (original) [34]. Ambient air pollutants in different units were uniformly converted into mass concentrations (μg/m^3^): (1) SO_2_: 1 ppb = 64/22.4 μg/m^3^; (2) NO_2_: 1 ppb = 46/22.4 μg/m^3^; (3) O_3_: 1 ppb = 48/22.4 μg/m^3^ (i.e., 22.4 is the molar volume of gas under standard conditions at 0 °C, 101.33 kPa) [35]. If eligible studies log-transformed the data of FBG before analysis, the extracted effect estimates were antilog-transformed. If the included study only reported regression coefficients, the coefficients were converted into percent changes per 10 μg/m^3^ increase in pollutant concentrations by applying the formula [β × 10 ÷ M] × 100%, where β denotes the regression coefficient and M denotes the average level of FBG [36].

The Cochrane Q statistical test (*p* < 0.05 considered significant) and the Standard I^2^ test were used to quantify the heterogeneity among all the included studies [35]. In most cases, a random-effects model was employed to pool changes in FBG associated with standardized increments in ambient air pollutant concentrations. However, when the heterogeneity among studies was small (i.e., I^2^ is less than 50%), a fixed-effect model was used instead [37]. In addition, per-specified subgroup and meta-regression analyses were performed to examine potential sources of heterogeneity. The subgroup criteria included the study area (Asia, Europe, and America), sample size (<5000 and ≥5000), participant type (general population and pregnant women), mean/median age of participant (≤18 years, 19–64 years and ≥65 years), female proportion (<50% and ≥50%), study design (cohort study and cross-sectional study), exposure assessment method (fixed site monitoring and model estimation), study quality (high and moderate), and number of controlled confounders (<10 and ≥10). The Q-tests were employed to evaluate the differences between subgroups [38]. Sensitivity analyses were performed to estimate the reliability of the findings by omitting one study at a time. To avoid potential bias caused by the artificial selection of exposure windows, sensitivity analyses were conducted to examine the influence of different exposure windows on the main results for each pollutant. For cases where the number of articles corresponding to different exposure windows was fewer than or equal to three, sensitivity analyses were not performed. Furthermore, we also examined the impact of including different studies conducted in the same population on the results in sensitivity analyses. Funnel plots, Egger’s test, and Begg’s test were applied to probe potential publication bias. All analyses were performed using the metafor package in R software (version 4.4.1), which can be used for meta-analysis, subgroup and regression analyses, and sensitivity analyses [39]. The pooled estimate with *p* < 0.05 (2-sided) was considered statistically significant.

## 3. Results

### 3.1. Literature Search and Characteristics of Included Studies

**Figure 1:**
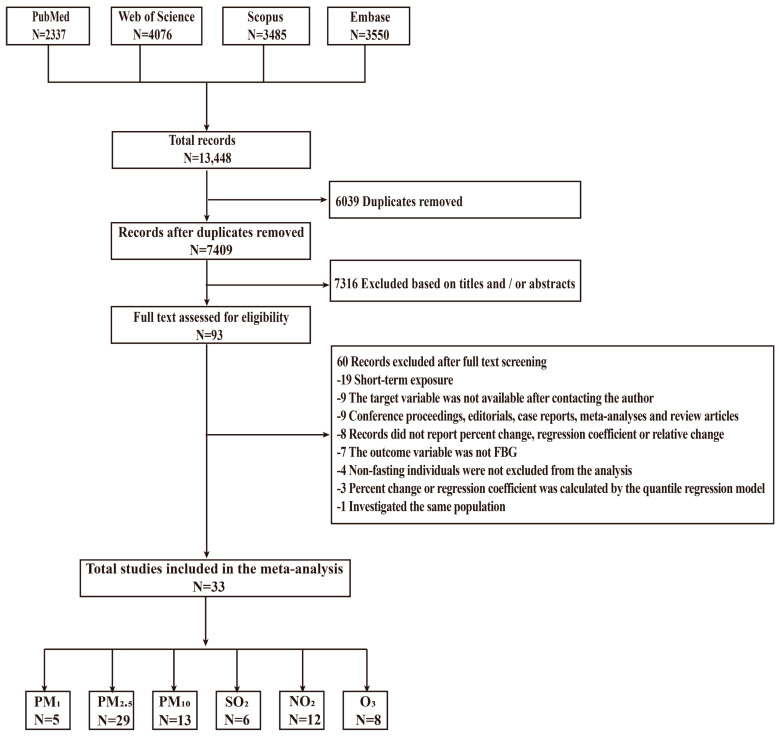
Flowchart of systematic literature search. Abbreviations: FBG, fasting blood glucose; NO_2_, nitrogen dioxide; O_3_, ozone; PM_1_, particulate matter with an aerodynamic diameter of ≤1 μm; PM_2.5_, particulate matter with an aerodynamic diameter of ≤2.5 μm; PM_10_, particulate matter with an aerodynamic diameter of 10 μm; SO_2_, sulfur dioxide.

After a systematic search in four designated databases, a total of 13,448 articles were retrieved. After excluding duplicates and articles that did not match by reading the titles and abstracts, a comprehensive analysis was conducted on 93 relevant articles. Ultimately, 33 articles were selected for inclusion in the meta-analysis (Figure 1). Table 1 summarizes the key details of the 33 studies. Among the 33 studies, there were 5, 29, 13, 6, 12, and 8 studies examining the associations of long-term exposures to PM_1_, PM_2.5_, PM_10_, SO_2,_ NO_2_, and O_3_ with FBG, respectively. Of all the studies included, 24 were carried out in Asia, 4 in Europe, and 5 in America, with sample sizes ranging from 113 to 20,076,032. The participant type involved the general population (covering all age groups) and pregnant women. The study design included cohort and cross-sectional studies. Ambient air pollutant data were mainly obtained from model estimation, followed by fixed-site monitoring. In terms of study quality, 16 studies were rated “high” quality, while the others were considered “moderate” quality (Table S3, Supplementary Materials).

### 3.2. Primary Meta-Analysis

**Figure 2:**
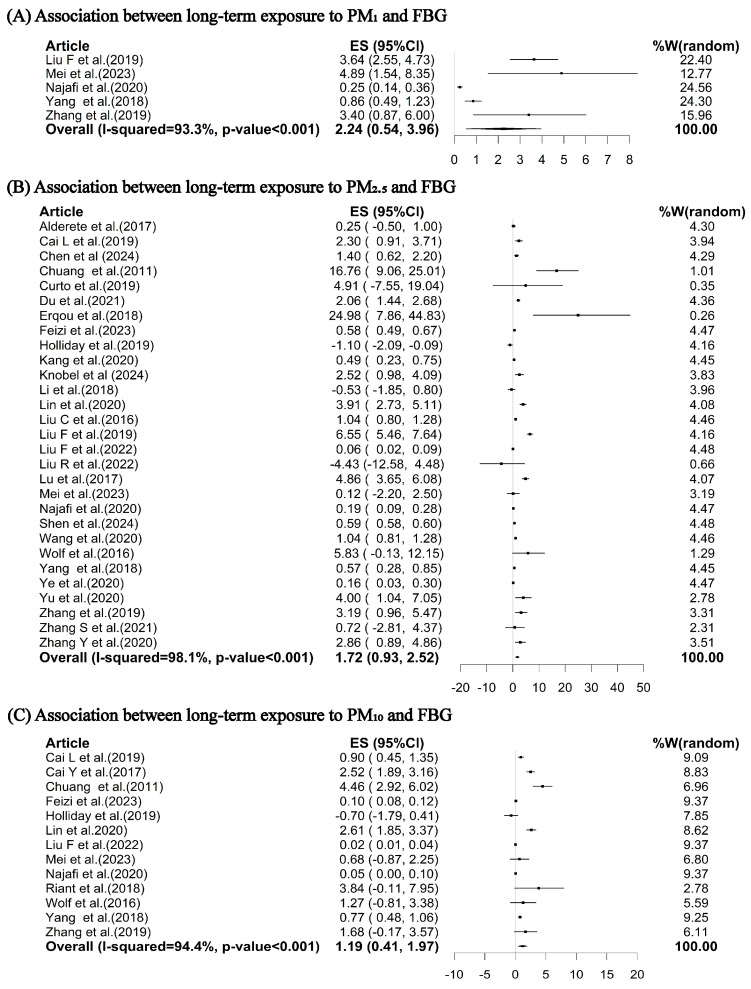
Pooled percent changes (%) and 95% confidence intervals in FBG per 10 μg/m^3^ increase in long-term exposures to PM_1_ (**A**) [8,10,12,56,63], PM_2.5_ (**B**) [8,10,12,24,25,26,27,40,41,43,44,45,46,47,48,50,51,52,53,55,56,58,59,60,61,62,63,65,66] and PM_10_ (**C**) [8,12,24,25,27,41,42,44,53,56,57,60,63]. ES represents the percent change; W represents the weighting of each included study; I-squared describes the percentage of variation in effect estimates due to heterogeneity rather than sampling error. The *p*-value is based on the Q-test. Abbreviations: FBG, fasting blood glucose; PM_1_, particulate matter with an aerodynamic diameter of ≤1 μm; PM_2.5_, particulate matter with an aerodynamic diameter of ≤2.5 μm; PM_10_, particulate matter with an aerodynamic diameter of ≤10 μm.

**Figure 3:**
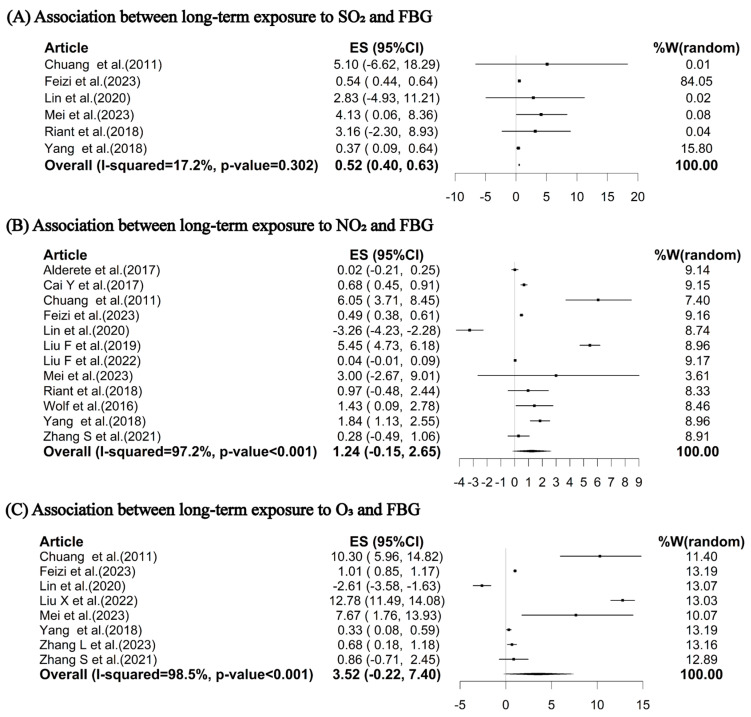
Pooled percent changes (%) and 95% confidence intervals in FBG per 10 μg/m^3^ increase in long-term exposures to SO_2_ (**A**) [8,12,24,27,44,57], NO_2_ (**B**) [8,10,12,24,27,40,42,44,53,57,60,65] and O_3_ (**C**) [8,12,24,27,44,54,64,65]. ES represents the percent change; W represents the weighting of each included study; I-squared describes the percentage of variation in effect estimates due to heterogeneity rather than sampling error. The *p*-value is based on the Q-test. Abbreviations: FBG, fasting blood glucose; NO_2_, nitrogen dioxide; O_3_, ozone; SO_2_, sulfur dioxide.

Our meta-analysis indicated that for each 10 μg/m^3^ increase in long-term exposure to PM_1_, PM_2.5_, PM_10_, and SO_2_, the pooled percent changes in FBG were 2.24% (95% CI: 0.54%, 3.96%), 1.72% (95% CI: 0.93%, 2.52%), 1.19% (95% CI: 0.41%, 1.97%), and 0.52% (95% CI: 0.40%, 0.63%), respectively (Figure 2A–C and Figure 3A). However, the associations of long-term exposures to ambient NO_2_ and O_3_ with FBG were insignificant: there were increases of 1.24% (95% CI: −0.15%, 2.65%) and 3.52% (95% CI: −0.22%, 7.40%) in FBG per 10 μg/m^3^ increase in NO_2_ and O_3_, respectively (Figure 3B, C).

### 3.3. Subgroup Analysis and Meta-Regression Analysis

Several potential sources of inter-study heterogeneity were identified by subgroup analysis and meta-regression analysis. For the association of long-term exposure to PM_1_ with FGB, the results of subgroup analysis revealed that participant type (*p* = 0.005) was a potential source of heterogeneity; specifically, for every 10 μg/m^3^ increase in PM_1_, FBG changed by 2.81% (95% CI: 1.04, 4.58) in the general population and by only 0.24% (95% CI: 0.13, 0.35) in pregnant women (Table S4, Supplementary Materials). For the association of long-term exposure to PM_10_ with FBG, the study area (*p* < 0.001) was found to be a source of heterogeneity in subgroup analysis; specifically, for every 10 μg/m^3^ increase in PM_10_, FBG changed by 1.12% (95% CI: 0.24, 2.00) in Asia, by 2.42% (95% CI: 1.83, 3.01) in Europe, and by −0.70% (95% CI: −1.81, 0.41) in America. Age (*p* < 0.001) was also found to be a source of heterogeneity in subgroup analysis for PM_10_; specifically, for every 10 μg/m^3^ increase in PM_10_, FBG changed by 0.94% (95% CI: 0.51, 1.37) in individuals with a mean/median age of ≤ 18 years, by 0.87% (95% CI: 0.11, 1.63) in individuals with a mean/median age of 19–64 years, and by 4.37% (95% CI: 2.88, 5.85) in individuals with a mean/median age of ≥ 65 years (Table S4, Supplementary Materials). Additionally, meta-regression analysis for PM_10_ also indicated that age (*p* = 0.034) may be a contributing factor to the observed heterogeneity. These findings align with those of the subgroup analyses, suggesting that FBG in individuals of age 65 years and above may be more susceptible to long-term exposure to PM_10_ (Table S5, Supplementary Materials). In addition, both subgroup analyses and meta-regression analyses found that the number of controlled confounders was a common source of heterogeneity for the effect estimates of PM_2.5_, PM_10_, and SO_2_ by subgroup analyses (Tables S4 and S5, Supplementary Materials). For subgroup analyses with the number of controlled confounders greater than or equal to 10, FBG changed by 1.10 (0.20, 2.01), 0.25 (−0.24, 0.73), and 0.50 (0.37, 0.64) for each 10 μg/m^3^ increase in the long-term exposure to PM_2.5_, PM_10_, and SO_2_, respectively. For subgroup analyses with the number of controlled confounders less than 10, FBG changed by 2.97 (1.37, 4.57), 1.82 (0.77, 2.87) and 3.67 (0.78, 6.55) per 10 μg/m^3^ increase in the long-term exposure to PM_2.5_, PM_10_ and SO_2_ exposures, respectively (Table S4, Supplementary Materials).

### 3.4. Publication Bias and Sensitivity Analyses

There may be certain degrees of publication bias in the studies about long-term exposure to PM_10_ and FBG, revealed by the funnel plot and Egger’s test, respectively, but Begg’s tests did not detect bias (Figure S1 and Table S6, Supplementary Materials). Meanwhile, we observed no significant publication bias among the studies examining the association between long-term exposures to PM_2.5_ and NO_2_ and FBG (Figure S1 and Table S6, Supplementary Materials). Considering the limited number of studies included (<10), publication bias for long-term exposures to PM_1_, SO_2_, and O_3_, and FBG could not be evaluated. Sensitivity analyses excluding individual studies each at a time did not result in obvious changes in the effect estimates (Table S7, Supplementary Materials). Additional sensitivity analyses for separate exposure windows showed that the pooled effect estimates at the main exposure window with the largest number of studies (1-year) for PM_2.5_, PM_10_, and O_3_ were consistent with the main results, but not for SO_2_ and NO_2_ (Table S8, Supplementary Materials). Furthermore, the results remained robust when the effect estimates from two different studies conducted in the same population were included in the analysis (Table S9, Supplementary Materials).

## 4. Discussion

This meta-analysis evaluated the associations between long-term exposures to major ambient air pollutants (PM_1_, PM_2.5_, PM_10_, SO_2_, NO_2_, and O_3_) and FBG based on data from a total of 33 eligible studies. Our analysis showed that FBG had a significantly positive association with ambient long-term exposures to ambient PMs (PM_1_, PM_2.5_, and PM_10_) and SO_2_, among which PMs showed stronger associations with FBG compared to the gaseous pollutant (SO_2_). On the other hand, our study did not reveal a statistical association of long-term exposures to ambient NO_2_ and O_3_ with FBG, although there was evidence of heterogeneity among the included studies. The main results underscore the potential adverse effects of long-term exposures to ambient PMs and SO_2_ on FBG.

Although the adverse impact of ambient air pollution on diabetes has become a global environmental health concern, there is less certainty about how long-term exposure to ambient air pollution affects FBG. While studies have generally demonstrated that long-term exposure to ambient air pollution leads to the risk of elevated FBG [8,24], there are still inconsistent reports. For example, Holliday et al. (2019) and Liu et al. (2022) did not observe any significant association of long-term exposures to PMs (PM_2.5_ and PM_10_) with FBG [25,26]. Chuang et al. (2011) and Lin et al. (2020) did not discover any association between SO_2_ and FBG either [27,44]. Ma et al. (2020) had the only meta-analysis so far reporting significant associations of long-term exposures to PM_2.5_ and PM_10_ with an elevated FBG, which is consistent with our results [28]. However, the analysis by Ma et al. (2020) was conducted only for PM_2.5_ and PM_10_, rather than a comprehensive meta-analysis for multiple major air pollutants (i.e., PM_1_, PM_2.5_, PM_10_, SO_2_, NO_2_, and O_3_) [28]. In this study, we separately investigated the potential effect of each major air pollutant long-term exposure on FBG. Moreover, our meta-analysis showed evidence for the potential adverse effects of long-term exposures to PM_1_ and SO_2_ on FBG, expanding the existing understanding of the association between ambient air pollution and FBG. Previous studies investigating the relationship between ambient air pollution and glucose metabolism have primarily focused on PM_2.5_ and PM_10_. However, PM_1_, as a sub-fraction of PM_2.5_, has a smaller particle size and is more likely to enter the circulatory system and exert systemic health effects [8,12]. Furthermore, Tian et al. (2023) performed a meta-analysis for the potential effect of ambient air pollution on HbA1c (a blood glucose indicator not affected by the diet or individual differences) and found that exposures to PM_2.5_ and PM_10_ were significantly associated with higher HbA1c levels, while the results for NO_2_ were not statistically significant, which aligns with the results of the present study, suggesting that exposure to air pollutants is linked to elevated glucose levels in the body [67]. To our knowledge, this is the first meta-analysis to comprehensively assess the potential effects of long-term exposures to both particulate and gaseous ambient air pollutants (i.e., PM_1_, PM_2.5_, PM_10_, SO_2,_ NO_2_, and O_3_) on FBG.

The pathophysiological mechanisms related to the associations of ambient air pollution with FBG are yet to be elucidated, but a synthesis of previous studies suggests several major potential biological pathways. One possible mechanism is that long-term exposure to air pollution can directly induce systemic inflammation and oxidative stress, which are linked to insulin resistance and beta-cell dysfunction, resulting in impaired glucose homeostasis [12,65]. Endothelial dysfunction is another mechanism hypothesizing that long-term exposure to air pollution affects endothelial function in humans and animals, resulting in decreased insulin sensitivity, as well as reduced peripheral blood glucose uptake [24,68]. Finally, long-term exposure to PM_2.5_ has been shown to down-regulate the expressions of several brown adipocyte-specific genes at the mRNA level and uncoupling protein 1 at the protein level in adipose depots, which may result in impaired glucose tolerance and insulin resistance, thus affecting blood glucose levels [21,69,70].

In this meta-analysis, several potential sources of heterogeneity among studies were identified based on subgroup and meta-regression analyses. In the analysis of the association of FBG with long-term exposure to PM_1_, it was observed that the general population exhibited a heightened susceptibility compared to pregnant women. However, previous studies have demonstrated that pregnant women have a special metabolic status during the mid-pregnancy period when FBG levels are measured. Due to the fast growth of the fetus, pregnant women may have excessive energy intake during pregnancy [71]. At the same time, pregnant women have an accelerated basal metabolic rate and are in a state of natural insulin resistance, rendering them more susceptible to glucose metabolism disorders and the hyperglycemic effects of air pollutants [26,72]. The reason for this anomalous result may be attributed to the limited number of subgroups of participant types (only one study of pregnant women) and the fact that no potential modification of the study results by participant type was observed in the meta-regression analysis. In the subgroup analysis of the association of FBG with long-term exposure to PM_10_, we observed that the pooled percent changes varied among the studies conducted in different study areas. The exposure levels of air pollutants, genetic susceptibility, lifestyle, and economic status of the population would be different over different study areas, and these variables may be potential factors influencing FBG levels. Nevertheless, the study area was not identified to be a potential modifier for the observed association in the meta-regression analysis, and such inconsistency between the subgroup and meta-regression analyses may be due to the limited number of subgroups of the study area. Furthermore, the association of FBG with long-term exposure to PM_10_ was stronger in individuals above 65 years of age compared to those below 65 years, which may be attributed to the fact that declines in physiological processes such as compromised clearance of particulates along the respiratory tract at older age, causing them to be more susceptible to glucose metabolism disturbances, thus affecting the effect estimates of air pollutants on FBG [65,73]. In the subgroup and meta-regression analyses of PM_1_ and PM_10_, there were limited numbers of studies in each category of participant type, age, and study area. More studies are needed to further validate the robustness of the results found in this study. In addition, subgroup analyses and meta-regression analyses also showed that the number of controlled confounders was also a source of heterogeneity. This may be because some studies adjusted for additional confounders, including exercise, dietary factors (e.g., fruit and vegetable intakes), family history of diabetes, or other factors that may influence the study outcome, in addition to controlling for common confounders (e.g., sex, age, body mass index, temperature, and relative humidity), whereas the other studies did not. In view that exercise increases exposure to air pollutants, and family history of diabetes and sugar intake can influence FBG measurements, it is understandable that the number of controlled confounders may be a source of heterogeneity.

In the sensitivity analyses for different exposure windows, the pooled effect estimates at the mostly used exposure window (i.e., 1-year) for PM_2.5_ and PM_10_ remained robust, corroborating the associations of long-term exposures to PM_2.5_ and PM_10_ with an elevated FBG in the main analysis. The association of SO_2_ with FBG became insignificant at the 1-year exposure window, which may be due to the limited number of studies (*n* = 4) available for the sensitivity analysis. Notably, the association between NO_2_ and FBG became significant at the 1-year exposure window in the sensitivity analysis, which is in contrast to the insignificant association between NO_2_ and FBG in the main analysis. This discrepancy could have been denoted by Lin et al. (2020) in the main analysis, which showed that NO_2_ was inversely associated with FBG at 6-month exposure window [27]. The results of the sensitivity analyses for different exposure windows warrant more research for validity in the future.

Although this study strictly followed the requirements of meta-analysis, several limitations should be considered. First, epidemiological studies on the association of long-term exposure to ambient air pollution with FBG are still limited, especially for smaller-PM (e.g., PM_1_) and gaseous pollutants (SO_2_, NO_2,_ and O_3_). Second, although we standardized the extracted effect estimates associated with a uniform increase of 10 μg/m^3^ in air pollutant concentrations for the included studies, the use of different statistical methods in the included studies may have a potential impact on the final results of the meta-analysis, but could not be evaluated. Third, most of the eligible studies used air pollution data from model estimation and fixed-site monitoring rather than monitoring data at the individual level, and this may affect the accuracy of the effect estimation in the original studies, which in turn affects the current meta-analysis. Therefore, it is recommended to increase the number of studies with exposure monitoring at the individual level to more accurately capture the actual air pollution exposure levels of the participants. Fourth, although we endeavored to contact the authors of studies that did not provide complete numerical data for quantitative transformation, several studies were still excluded because the authors could not be reached for quantitative result data, which might introduce a certain degree of selection bias and thus affect the results to some extent. Nevertheless, most of the included studies did not adjust for noise or poverty level, both of which have been associated with FBG in previous studies [74,75,76,77]. Our study was unable to evaluate whether the adjustment for these factors may have any impact on the observed results.

## 5. Conclusions

In conclusion, our study comprehensively assessed the association of long-term exposure to ambient air pollution with FBG and found that FBG was positively associated with long-term exposures to PM_1_, PM_2.5_, PM_10_, and SO_2_. Subgroup analysis identified elderly individuals to be more vulnerable to air-pollution-associated adverse effects in FBG levels. Sensitivity analysis showed that NO_2_ was positively associated with FBG at the 1-year exposure window. Our study expands the current understanding of the association of ambient air pollution with the risk of diabetes, which could provide additional insights for disease prevention.

## Figures and Tables

**Table 1:** Main characteristics of the studies included in the meta-analysis.

Author	Study Area	Study Period	Sample Size	Participant Type	Age	Female Proportion (%)	Study Design	Exposure Assessment Method	Study Quality	Exposure Window	Pollutants	Incremental Scale (μg/m^3^)	Percent Changes (%) and 95% CIs
Alderete et al. [40] (2017)	America	2001 to 2012	314	General population	11.4 ± 1.7 ^a^	70.4	Cohort study	Fixed-site monitoring	High	1 y average	PM_2.5_	4	0.1 (−0.2, 0.4)
											NO_2_	10.71	0.02 (−0.2, 0.3)
Cai L et al. [41] (2019)	China	2016 to 2017	4234	General population	9.1 ± 1.8 ^a^	46.6	Cross-sectional study	Model estimation	Moderate	6 m average	PM_2.5_	10	2.3 (1.0, 3.8)
											PM_10_	10	0.9 (0.5, 1.4)
Cai Y et al. [42] (2017)	Netherland	2006 to 2013	93,277	General population	44.9 ± 12.3 ^a^	59	Cross-sectional study	Model estimation	Moderate	1 y average	PM_10_	2.4	0.6 (0.4,0.7)
											NO_2_	8.8	0.6 (0.4, 0.8)
Chen et al. [43] (2024)	China	2017 to 2018	834	pregnant women	30.3 ± 4.8 ^a^	100	Cohort study	Model estimation	High	6 m average	PM_2.5_	12.92	0.08 (0.04, 0.13)
Chuang et al. [44] (2011)	China	2000	1022	General population	69.2 ± 8.7 ^a^	42.3	Cross-sectional study	Fixed-site monitoring	Moderate	1 y average	PM_2.5_	48	22.88 (14.93, 30.82)
											PM_10_	20.42	36.55 (19.20, 53.90)
											SO_2_	19.18	21.10 (12.03, 30.17)
											NO_2_	26.35	17.03 (10.37, 23.69)
											O_3_	9.09	4.95 (−7.05, 16.95)
Curto et al. [45] (2019)	India	2010 to 2012	5065	General population	37.5 ± 13.4 ^a^	46	Cross-sectional study	Model estimation	Moderate	1 y average	PM_2.5_	1	0.48 (−0.78, 1.76)
Du et al. [46] (2021)	China	2017 to 2019	5852	General population	64.6 ± 13.6 ^a^	50.5	Cross-sectional study	Model estimation	Moderate	1 y average	PM_2.5_	28.8	0.35 (0.25, 0.46)
Erqou et al. [47] (2018)	North America	2012 to 2013	1499	General population	59 ± 8 ^a^	66	Cohort study	Model estimation	High	1 y average	PM_2.5_	1.5	3.71 (0.99, 6.42)
Feizi et al. [24] (2023)	Iran	2012 to 2018	3826	General population	43.1 ± 6.3 ^a^	70.4	Cohort study	Fixed-site monitoring	High	1 y average	PM_2.5_	1	0.060 (0.051, 0.070)
											PM_10_	1	0.010 (0.008, 0.012)
											SO_2_	1	0.056 (0.046, 0.067)
											NO_2_	1	0.050 (0.040, 0.063)
											O_3_	1	0.102 (0.088, 0.120)
Holliday et al. [25] (2019)	America	1993 to 2004	3915	General population	62.7 ^b^	100	Cohort study	Model estimation	High	1 y average	PM_10_	10	−0.7 (−1.8, 0.4)
											PM_2.5_	10	−1.1 (−2.1, −0.1)
Kang et al. [48] (2020)	China	2013 to 2015	4783	Pregnant women	28.5 ± 3.3 ^a^	100	Cohort study	Model estimation	High	6 m average	PM_2.5_	10	0.382 (0.179, 0.586)
Kang et al. [49] (2023) ^d^	China	2015 to 2017	38,442	General population	55.6 ± 12.2 ^a^	61	Cohort study	Model estimation	High	3 y average	PM_2.5_	1	0.01 (0.01, 0.02)
Knobel et al. [50] (2024)	America	2004 to 2019	81,599	General	51.9 ± 9.3	15	Cohort study	Model estimation	High	6 m average	PM_2.5_	3.09	0.78 (0.30, 1.26)
Li et al. [51] (2018)	America	2003	5958	General population	51 ^b^	55	Cohort study	Model estimation	High	1 y average	PM_2.5_	1.5	−0.08 (−0.28, 0.12)
Lin et al. [27] (2020)	China	2015 to 2019	12,842	Pregnant women	18–45	100	Cohort study	Fixed-site monitoring	High	6 m average	PM_2.5_	10	0.18 (0.13, 0.24)
											PM_10_	10	0.12 (0.08, 0.15)
											SO_2_	10	0.13 (−0.24, 0.50)
											NO_2_	10	−0.15 (−0.19, −0.10)
											O_3_	10	−0.12 (−0.16, −0.07)
Liu C et al. [52] (2016)	China	2011 to 2012	11,847	General population	59.3 ± 10.6 ^a^	52	Cross-sectional study	Model estimation	Moderate	1 y average	PM_2.5_	41.1	0.26 (0.20, 0.32)
Liu F et al. [10] (2019) ^d^	China	2015 to 2017	39,191	General population	55.6 ± 12.2 ^a^	60.6	Cohort study	Model estimation	High	3 y average	PM_1_	1	0.020 (0.014, 0.026)
											PM_2.5_	1	0.036 (0.030, 0.042)
											NO_2_	1	0.030 (0.026, 0.034)
Liu F et al. [53] (2022)	China	2009 to 2011	9638	General population	60.3 ± 9.7 ^a^	54.1	Cross-sectional study	Model estimation	Moderate	2 y average	PM_2.5_	10	0.061 (0.028, 0.096)
											PM_10_	10	0.025 (0.007, 0.044)
											NO_2_	10	0.044 (−0.009, 0.097)
Liu R et al. [26] (2022)	China	2016 to 2017	113	Pregnant women	31.2 ± 3.1 ^a^	100	Cohort study	Model estimation	High	6-m average	PM_2.5_	10	−4.43 (−12.92, 4.07)
Liu X et al. [54] (2022)	China	2015 to 2017	39,192	General population	18–79	61.95	Cross-sectional study	Model estimation	Moderate	3 y average	O_3_	4.04	0.286 (0.257, 0.315)
Lu et al. [55] (2017) ^e^	China	2006 to 2014	3589	Pregnant women	31.4 ± 4.5 ^a^	100	Cross-sectional study	Fixed-site monitoring	Moderate	6 m average	PM_2.5_	14.45	5.87 (4.40, 7.34)
										1 y average		6.1	3.35 (2.42, 4.28)
Mei et al. [12] (2023)	China	2018 to 2020	4235	General population	54.2 ± 14.6 ^a^	50.32	Cross-sectional study	Model estimation	Moderate	1 y average	PM_1_	10	4.89 (1.54, 8.35)
											PM_2.5_	10	0.12 (−2.21, 2.49)
											PM_10_	10	0.68 (−0.86, 2.26)
											SO_2_	10	3 (−2.67, 9.01)
											NO_2_	10	4.13 (0.06, 8.36)
											O_3_	10	7.67 (1.75, 13.92)
Najafi et al. [56] (2020)	Iran	2019	250	Pregnant women	28 ± 9 ^c^	100	Cross-sectional study	Model estimation	Moderate	1 y average	PM_1_	40.8	0.69 (0.38, 1.00)
											PM_2.5_	47.4	0.61 (0.29, 0.93)
											PM_10_	52.9	0.19 (0.01, 0.37)
Riant et al. [57] (2018)	France	2011 to 2013	2741	General population	40–65	>50	Cross-sectional study	Model estimation	Moderate	1 y average	NO_2_	5	0.0046 (−0.0024, 0.0115)
Shen et al. [58] (2024) ^e^	China	2010 to 2015	20,076,032	General population	27.0 ± 4.8 ^a^	100	Cross-sectional	Model estimation	Moderate	1 y average	PM_2.5_	26	0.075 (0.074, 0.076)
										2 y average		27	0.077 (0.076, 0.078)
										3 y average		27	0.078 (0.077, 0.079)
											PM_10_	2	0.0073 (−0.0003, 0.0150)
											SO_2_	2	0.006 (−0.0047, 0.0166)
Wang et al. [59] (2020)	China	2007 to 2013	16,489	General population	6–17	48.9	Cross-sectional study	Model estimation	Moderate	7 y average	PM_2.5_	10	0.049 (0.038, 0.060)
Wolf et al. [60] (2016)	Germany	2006 to 2008	2944	General population	56.2 ± 13.1 ^a^	51.6	Cohort study	Model estimation	High	1 y average	PM_2.5_	2.8	1.6 (0.0, 3.3)
											PM_10_	7.9	1.0 (0.7, 2.6)
											NO_2_	11.9	1.7 (0.1, 3.3)
Yang et al. [8] (2018)	China	2006 to 2008	24,845	General population	45.0 ± 13.5 ^a^	47.3	Cross-sectional study	Model estimation	Moderate	3 y average	PM_1_	15	0·07 (0·04, 0·10)
											PM_2.5_	26	0·08 (0·04, 0·12)
											PM_10_	19	0·08 (0·05, 0·11)
											SO_2_	20	0·04 (0·01, 0·07)
											NO_2_	9	0·09 (0·06, 0·13)
											O_3_	22	0·04 (0·01, 0·07)
Ye et al. [61] (2020)	China	2013 to 2016	3967	Pregnant women	28.2 ± 3.5 ^a^	100	Cohort study	Model estimation	High	1 y average	PM_2.5_	23.3	0.38 (0.07, 0.70)
Yu et al. [62] (2020)	Indonesia	2013 to 2016	469	General population	14–18	42.6	Cross-sectional study	Model estimation	Moderate	4 y average	PM_2.5_	1	0.34 (0.08, 0.59)
Zhang et al. [63] (2019)	China	2012	11,814	General population	11.7 ± 3.2 ^a^	50.6	Cross-sectional study	Model estimation	Moderate	1 y average	PM_1_	10	0.160 (0.039, 0.280)
											PM_2.5_	10	0.150 (0.044, 0.256)
											PM_10_	10	0.079 (−0.009, 0.167)
Zhang L et al. [64] (2023)	China	2017 to 2018	7834	Pregnant women	18–45	100	Cohort study	Model estimation	High	6 m average	O_3_	30.42	0.162 (0.436, 2.804)
Zhang S et al. [65] (2021)	Germany	1999 to 2014	6008	General population	25–74	>50	Cohort study	Model estimation	High	1 y average	PM_2.5_	1.4	0.1 (−0.4, 0.6)
											NO_2_	7.1	0.2 (−0.4, 0.7)
											O_3_	3.5	0.3 (−0.3, 0.8)
Zhang Y et al. [66] (2020) ^e^	China	2005 to 2016	1449	General population	83 ± 12 ^a^	52.6	Cohort study	Model estimation	High	1 y average	PM_2.5_	10	0.146 (0.045, 0.248)
										2 y average	PM_2.5_	10	0.109 (0.023,0.195)
										3 y average	PM_2.5_	10	0.146 (0.045, 0.248)

^a^ Mean ± standard deviation (SD). ^b^ Mean. ^c^ Median ± interquartile range (IQR). ^d^ In cases where multiple studies were conducted on the same population, the study by Liu F et al. (2019) that investigated a higher number of ambient air pollutants was included in the main analysis, and the study by Kang et al. (2023) was included in the sensitivity analysis (see results in the Supplemental Materials) [49]. ^e^ The effect estimate from the exposure window with the most significant effect value (smallest *p*-value) was selected for the main meta-analysis, and the effect estimates for the remaining time windows were used for sensitivity analysis. Abbreviations: CI, confidence interval; NO_2_, nitrogen dioxide; O_3_, ozone; PM_1_, particulate matter with an aerodynamic diameter of ≤1 μm; PM_2.5_, particulate matter with an aerodynamic diameter of ≤2.5 μm; PM_10_, particulate matter with an aerodynamic diameter of ≤10 μm; SO_2_, sulfur dioxide.

## Data Availability

The data that support the findings of this study are available from the corresponding author upon reasonable request.

## Supplementary Materials

The following supporting information can be downloaded at: https://www.mdpi.com/article/10.3390/toxics12110792/s1, Figure S1: Funnel plots of publication bias for the association between long-term exposure to ambient air pollution and FBG; Table S1: Detailed search strategy of the meta-analysis; Table S2: Explanatory file for Effective Public Health Practice Project (EPHPP) quality assessment tool; Table S3: Quality assessment using the EPHPP quality assessment tool for the included studies; Table S4: Subgroup analysis for the association between long-term exposure to ambient air pollution and FBG; Table S5: Meta-regression analysis for the association between long-term exposure to ambient air pollution and FBG; Table S6: Publication bias of the included studies; Table S7: Results of sensitivity analyses omitting one study each at a time; Table S8: Results of sensitivity analyses for different exposure window; Table S9: Results of sensitivity analyses replacing studies with the same population.
