# Supplementary material for: Association Between Long-Term Exposure to Ambient Air Pollution and Fasting Blood Glucose: A Systematic Review and Meta-Analysis
Source: Toxics. 2024 Oct 30;12(11):792. doi: 10.3390/toxics12110792 (PMC11598464; doi:10.3390/toxics12110792)
Supplement: Supplementary file 1 [file toxics-12-00792-s001.zip › toxics-3210855-supplementary.pdf]

Supplementary Material

Association between long-term exposure to ambient air pollution and fasting blood glucose: a systematic review and meta-analysis

Tong Wu, Yang Lan, Ge Li, Kai Wang, Yu You, Jiaqi Zhu, Lihua Ren, Shaowei Wu

Table of contents

Table S1. Detailed search strategy of the meta-analysis.
Table S2. Explanatory file for Effective Public Health Practice Project (EPHPP) quality assessment tool.
Table S3. Quality assessment using the EPHPP quality assessment tool for the included studies.
Table S4. Subgroup analysis for the association between long-term exposure to ambient air pollution and FBG.
Table S5. Meta-regression analysis for the association between long-term exposure to ambient air pollution and FBG.
Table S6. Publication bias of the included studies.
Table S7. Results of sensitivity analyses omitting one study each at a time.
Table S8. Results of sensitivity analyses for different exposure window.
Table S9. Results of sensitivity analyses replacing studies with the same population.

Figure of content

Fig. S1. Funnel plots of publication bias for the association between long-term exposure to ambient air pollution and FBG.

Table S1. Detailed search strategy of the meta-analysis.

Database	Literature search strategy
1. PubMed	#1"Air Pollution"[Mesh] OR "Air Pollution" OR "Air pollutants" OR" Particulate Matter" OR "PM" OR "PM_1" OR "$PM_{2.5}$" OR "PM_{10}" OR "Particles" OR "Sulfur dioxide" OR "Nitrogen dioxide" OR "Ozone" OR "Gaseous air pollutant"<br>#2"Blood Glucose"[Mesh] OR "Blood Glucose" OR "Blood sugar" OR "Fasting blood glucose" OR "Fasting plasma glucose"<br>#3 (1 AND 2)
2. Web of Science (WOS)	#1 TS= ("Air Pollution" OR "Air pollutants" OR" Particulate Matter" OR "PM" OR "PM_1" OR "$PM_{2.5}$" OR "PM_{10}" OR "Particles" OR "Sulfur dioxide" OR "Nitrogen dioxide" OR "Ozone" OR "Gaseous air pollutant")<br>#2 TS= ("Blood Glucose" OR "Blood Sugar " OR "Fasting blood glucose" OR "Fasting plasma glucose")<br>#3 (1 AND 2)
3. Scopus	#1 TITLE-ABS-KEY ("Air Pollution" OR "Air pollutants" OR "Particulate Matter" OR "PM" OR "PM_1" OR "$PM_{2.5}$" OR "PM_{10}" OR "Particles" OR "Sulfur dioxide" OR "Nitrogen dioxide" OR "Ozone" OR "Gaseous air pollutant")<br>#2 TITLE-ABS-KEY ("Blood Glucose" OR "Blood Sugar" OR "Fasting blood glucose" OR "Fasting plasma glucose")<br>#3 (1 AND 2)
4. EMBASE	#1 ('air pollution'/exp OR 'air pollution' OR 'air pollutants' OR 'particulate matter'/exp OR 'particulate matter' OR 'pm' OR 'pm_1'/exp OR 'pm_1' OR '$pm_{2.5}$'/exp OR '$pm_{2.5}$' OR 'pm_{10}'/exp OR 'pm_{10}' OR 'particles' OR 'sulfur dioxide'/exp OR 'sulfur dioxide' OR 'nitrogen dioxide'/exp OR 'nitrogen dioxide' OR 'ozone'/exp OR 'ozone' OR 'gaseous air pollutant')<br>#2 ('blood glucose'/exp OR 'blood glucose' OR 'blood sugar'/exp OR 'blood sugar' OR 'fasting blood glucose'/exp OR 'fasting blood glucose' OR 'fasting plasma glucose'/exp OR 'fasting plasma glucose')<br>#3 (1 AND 2)

Table S2. Explanatory file for Effective Public Health Practice Project (EPHPP) quality assessment tool.

Component ratings	Details
A. Selection bias	Good: (Q1 is 1) and (Q2 is 1).<br>Moderate: (Q1 is 1 or 2) and (Q2 is 2).<br>Poor: (Q1 is 3); or (Q2 is 3).
Q1. Are the individuals selected to participate in the study likely to be representative of the target population?	
1 Very likely	Randomly selected from a comprehensive list of individuals in the target population.
2 Somewhat likely	Referred from a source (e.g. clinic) in a systematic manner.
3 Not likely	Self-referred.
Q2. What percentage of selected individuals agreed to participate?	
1 80 - 100% agreement<br>2 60 - 79% agreement<br>3 Less than 60% agreement<br>4 Not applicable	Refers to the percentage of subjects in the control and intervention groups that agreed to participate in the study before they were assigned to intervention or control groups.
B. Study design	Good: (Q1 is 1).<br>Moderate: (Q1 is 2).<br>Weak: (Q1 is 3).
Q1. Indicate the study design	
1 Strong design	Randomized controlled trials (RCTs) and controlled clinical trials (CCTs).
2 Moderate design	Cohort or case-crossover designs.
3 Weak design	Cross-sectional or time-series designs.
C. Confounders	Good: (Q1 is 2); or (Q2 is 1).<br>Moderate: (Q1 is 1) and (Q2 is 2).<br>Poor: (Q1 is 1) and (Q2 is 3).
Q1. Were there important differences between groups prior to the intervention?	
1 Yes	Important differences were found.
2 No	No important differences were found.
Q2. If yes, indicate the percentage of relevant confounders that were controlled (either in the design (e.g. stratification, matching) or analysis)?	
1 80-100%	Cross-sectional and cohort: meteorological variables and demographic characteristics.<br>Case-crossover and time-series: meteorological variables and time-effects.
2 60-79%	Cross-sectional and cohort: meteorological variables or demographic characteristics.<br>Case-crossover and time-series: meteorological variables or time-effects.

Component ratings	Details
3 Less than 60% or none	Not controlled.
D. Blinding	Good: (Q1 is 2) and (Q2 is 2).<br>Moderate: (Q1 is 2) or (Q2 is 2).<br>Poor: (Q1 is 1) and (Q2 is 1).
Q1. Was (were) the outcome assessor(s) aware of the intervention or exposure status of participants?	
1 Yes	The outcome assessors were aware of the exposure status of participants.
2 No	The outcome assessors were not aware of the exposure status of participants.
Q2. Were the study participants aware of the research question?	
1 Yes	The participants were aware of the exposure status of participants.
2 No	The participants were not aware of the exposure status of participants.
E. Data collection methods	Good: (Q1 is 1) and (Q2 is 1).<br>Moderate: (Q1 is 1) and (Q2 is 2).<br>Poor: (Q1 is 2).
Q1. Were data collection tools shown to be valid?	
1 Yes	The blood sample was detected by professionals and trained investigators.
2 No	The method for blood sample assay was not introduced.
Q2. Were data collection tools shown to be reliable?	
1 Yes	Methods had been acknowledged or with evidence from previous researches.
2 No	Methods were not clearly addressed.
F. Withdrawals and drop-outs	Good: (Q1 is 1) and (Q2 is 1).<br>Moderate: (Q2 is 2).<br>Poor: (Q2 is 3); or (Q1 is 2).<br>Not Applicable: (Q1 is 3); or (Q2 is 4).
Q1. Were withdrawals and drop-outs reported in terms of numbers and/or reasons per group?	
1 Yes	Describe both the numbers and reasons for withdrawals and drop-outs.
2 No	Either the numbers or reasons for withdrawals and drop-outs are not reported.
3 Not Applicable	Not follow-up data.
Q2. Indicate the percentage of participants completing the study. (If the percentage differs by groups, record the lowest).	
1 80 -100%<br>2 60 - 79%<br>3 less than 60%<br>4 Not Applicable	The percentage of participants completing the study refers to the % of subjects remaining in the study at the final data collection period in all groups (i.e. control and intervention groups).

Overall ratings: 1) High- no “Poor” ratings. 2) Moderate- one “Poor” rating. 3) Poor– two or more “Poor” ratings.

Table S3. Quality assessment using the Effective Public Health Practice Project (EPHPP) quality assessment tool for the included studies.

Author	Selection	Design	Confounder	Blinding	Data	Withdraw	Overall
Alderete et al. (2017)	High	Moderate	High	High	High	High	High
Cai L et al. (2019)	High	Poor	High	High	High	Not Applicable	Moderate
Cai Y et al. (2017)	High	Poor	High	High	High	Not Applicable	Moderate
Chen et al. (2024)	High	Moderate	High	High	High	High	High
Chuang et al. (2011)	High	Poor	High	High	High	Not Applicable	Moderate
Curto et al. (2019)	Moderate	Poor	High	High	High	Not Applicable	Moderate
Du et al. (2021)	High	Poor	High	High	High	Not Applicable	Moderate
Erqou et al. (2018)	High	Moderate	High	High	High	—	High
Feizi et al. (2023)	Moderate	Moderate	High	High	High	High	High
Holliday et al. (2019)	High	Moderate	High	High	High	High	High
Kang et al. (2020)	High	Moderate	High	High	High	High	High
Li et al. (2018)	High	Moderate	High	High	High	High	High
Lin et al. (2020)	Moderate	Moderate	High	High	High	High	High
Liu C et al. (2016)	High	Poor	High	High	High	Not Applicable	Moderate
Liu F et al. (2019)	High	Moderate	High	High	High	High	High
Liu F et al. (2022)	High	Poor	High	High	High	Not Applicable	Moderate
Liu R et al. (2022)	Moderate	Moderate	High	High	High	High	High
Liu X et al. (2022)	High	Poor	High	High	High	Not Applicable	Moderate
Lu et al. (2017)	High	Poor	High	High	High	Not Applicable	Moderate

Author	Selection	Design	Confounder	Blinding	Data	Withdraw	Overall
Mei et al. (2023)	High	Poor	High	High	High	Not Applicable	Moderate
Najafi et al. (2020)	Moderate	Poor	High	High	High	Not Applicable	Moderate
Pablo et al. (2024)	High	Moderate	High	High	High	High	High
Riant et al. (2018)	High	Poor	High	High	High	Not Applicable	Moderate
Shen et al (2024)	High	Poor	High	High	High	Not Applicable	Moderate
Wang et al. (2020)	High	Poor	High	High	High	Not Applicable	Moderate
Wolf et al. (2016)	High	Moderate	High	High	High	High	High
Yang et al. (2018)	High	Poor	High	High	High	Not Applicable	Moderate
Ye et al. (2020)	High	Moderate	High	High	High	High	High
Yu et al. (2020)	High	Poor	High	High	High	Not Applicable	Moderate
Zhang et al. (2019)	High	Poor	High	High	High	Not Applicable	Moderate
Zhang L et al. (2023)	Moderate	Moderate	High	High	High	High	High
Zhang S et al. (2021)	High	Moderate	High	High	High	High	High
Zhang Y et al. (2020)	High	Moderate	High	High	High	High	High

Table S4. Subgroup analysis for the association between long-term exposure to ambient air pollution and FBG.

Grouping variable	Grouping criterion	No. of studies	Pooled %-change (95% CI)	*P*-value	I^2	*P*-value for heterogeneity	*P*-value for subgroup difference
Study area							
$PM_{2.5}$	Asia	22	1.91 (1.03, 2.78)	<0.001	98.57%	<0.001	0.276
	America	5	0.47 (-1.12, 2.06)	0.561	83.85%	<0.001	
	Europe	2	2.64 (-2.09, 7.38)	0.274	50.87%	0.154	
PM_{10}	Asia	9	1.12 (0.24, 2.00)	0.012	94.68%	<0.001	<0.001
	America	1	-0.70(-1.81, 0.41)	0.214	—	—	
	Europe	3	2.42 (1.83, 3.01)	<0.001	0.00%	0.422	
SO_2	Asia	5	0.51 (0.40, 0.63)	<0.001	22.64%	0.270	0.350
	Europe	1	3.11 (-2.33, 8.55)	0.263	—	—	
NO_2	Asia	7	1.75 (-0.72, 4.22)	0.164	98.35%	<0.001	<0.001
	America	1	0.02 (-0.21, 0.25)	0.875	—	—	
	Europe	4	0.67 (0.46, 0.89)	<0.001	0.00%	0.502	
O_3	Asia	7	3.87 (-0.32, 8.06)	0.070	98.68%	<0.001	0.187
	Europe	1	0.86 (-0.71, 2.42)	0.285	—	—	
Sample size							
PM_1	<5000	2	2.21 (-2.19, 6.61)	0.325	86.62%	0.006	0.926

Grouping variable	Grouping criterion	No. of studies	Pooled %-change (95% CI)	*P*-value	I^2	*P*-value for heterogeneity	*P*-value for subgroup difference
	≥5000	3	2.43 (0.53, 4.34)	0.012	92.26%	<0.001	
$PM_{2.5}$	<5000	17	1.89 (0.71, 3.07)	0.002	91.97%	<0.001	0.720
	≥5000	12	1.59 (0.45, 2.73)	0.006	99.13%	<0.001	
PM_{10}	<5000	8	1.02 (-0.12, 2.16)	0.079	87.14%	<0.001	0.587
	≥5000	5	1.45 (0.40, 2.51)	0.007	97.03%	<0.001	
SO_2	<5000	4	1.90 (-0.54, 4.35)	0.127	31.49%	0.223	0.222
	≥5000	2	0.37 (0.10, 0.64)	0.008	0.00%	0.545	
NO_2	<5000	6	1.63 (-0.07, 3.34)	0.060	87.32%	<0.001	0.566
	≥5000	6	0.81 (-1.41, 3.03)	0.473	98.43%	<0.001	
O_3	<5000	3	5.67 (0.06, 11.28)	0.048	91.42%	<0.001	0.369
	≥5000	5	2.24 (-2.70, 7.19)	0.374	99.06%	<0.001	
Participant type							
PM_1	General population	4	2.81 (1.04, 4.58)	0.002	90.16%	<0.001	0.005
	Pregnant women	1	0.24 (0.13, 0.35)	<0.001	—	—	
$PM_{2.5}$	General population	22	1.78 (0.84, 2.73)	<0.001	98.42%	<0.001	0.821
	Pregnant women	7	1.57 (0.01, 3.13)	0.048	94.59%	<0.001	

Grouping variable	Grouping criterion	No. of studies	Pooled %-change (95% CI)	*P*-value	I^2	*P*-value for heterogeneity	*P*-value for subgroup difference
PM_{10}	General population	11	1.17 (0.31, 2.02)	0.008	94.17%	<0.001	0.929
	Pregnant women	2	1.29 (-1.19, 3.76)	0.308	97.74%	<0.001	
SO_2	General population	5	0.51 (0.40, 0.63)	<0.001	30.51%	0.218	0.570
	Pregnant women	1	2.79 (-5.05, 10.63)	0.486	—	—	
NO_2	General population	11	1.62 (0.42, 2.83)	0.008	97.11%	<0.001	<0.001
	Pregnant women	1	-3.32 (-4.33, -2.31)	<0.001	—	—	
O_3	General population	6	5.03 (0.77, 9.30)	0.021	98.76%	<0.001	0.029
	Pregnant women	2	-0.95 (-4.21, 2.30)	0.566	97.05%	<0.001	
Mean/median age							
PM_1	≤18y	1	3.35 (0.87, 5.83)	0.008	—	—	0.415
	19-64y	4	2.03 (0.07, 4.00)	0.042	94.43%	<0.001	
$PM_{2.5}$	≤18y	5	1.71 (0.51, 2.91)	0.005	72.94%	0.005	0.490
	19-64y	22	1.43 (0.53, 2.32)	0.002	98.55%	<0.001	
	≥65y	2	8.72 (-3.68, 21.11)	0.168	91.85%	<0.001	
PM_{10}	≤18y	2	0.94 (0.51, 1.37)	<0.001	0.00%	0.425	<0.001
	19-64y	10	0.87 (0.11, 1.63)	0.025	94.61%	<0.001	

Grouping variable	Grouping criterion	No. of studies	Pooled %-change (95% CI)	*P*-value	I^2	*P*-value for heterogeneity	*P*-value for subgroup difference
	≥65y	1	4.37 (2.88, 5.85)	<0.001	—	—	
SO_2	19-64y	5	0.51 (0.40, 0.63)	<0.001	27.21%	0.240	0.459
	≥65y	1	4.98 (-6.85, 16.80)	0.409	—	—	
NO_2	≤18y	1	0.02 (-0.21, 0.25)	0.875	—	—	<0.001
	19-64y	10	0.94 (-0.46, 2.34)	0.188	97.53%	<0.001	
	≥65y	1	5.88 (3.64, 8.11)	<0.001	—	—	
O_3	19-64y	7	2.63 (-1.12, 6.38)	0.169	98.62%	<0.001	0.011
	≥65y	1	9.80 (5.79, 13.82)	<0.001	—	—	
Female proportion							
PM_1	<50%	1	0.85 (0.49, 1.22)	<0.001	—	—	0.086
	≥50%	4	2.69 (0.62, 4.75)	0.011	94.14%	<0.001	
$PM_{2.5}$	<50%	7	2.60 (-0.06, 5.27)	0.056	83.17%	<0.001	0.502
	≥50%	22	1.64 (0.73, 2.55)	<0.001	98.56%	<0.001	
PM_{10}	<50%	3	1.90 (-0.29, 4.10)	0.090	90.80%	<0.001	0.426
	≥50%	10	0.95(0.14, 1.77)	0.022	93.85%	<0.001	
SO_2	<50%	2	0.37 (0.09, 0.64)	0.008	0.00%	0.434	0.227

Grouping variable	Grouping criterion	No. of studies	Pooled %-change (95% CI)	*P*-value	I^2	*P*-value for heterogeneity	*P*-value for subgroup difference
	≥50%	4	1.78 (-0.49, 4.05)	0.125	27.77%	0.245	
NO_2	<50%	3	2.41 (-0.85, 5.68)	0.147	95.79%	0.001	0.394
	≥50%	9	0.84 (-0.72, 2.40)	0.290	97.67%	<0.001	
O_3	<50%	2	4.85 (-4.42, 14.12)	0.305	95.30%	<0.001	0.732
	≥50%	6	3.06 (-1.32, 7.43)	0.171	98.78%	<0.001	
Study design							
PM_1	Cross-sectional study	4	1.81 (-0.07, 3.69)	0.059	86.92%	<0.001	0.110
	Cohort study	1	3.57 (2.52, 4.62)	<0.001	—	—	
$PM_{2.5}$	Cross-sectional study	15	1.61 (0.38, 2.83)	0.010	93.83%	<0.001	0.852
	Cohort study	14	1.76 (0.79, 2.72)	<0.001	98.98%	<0.001	
PM_{10}	Cross-sectional study	9	1.37 (0.42, 2.32)	0.005	94.36%	<0.001	0.513
	Cohort study	4	0.79 (-0.69, 2.26)	0.297	93.48%	<0.001	
SO_2	Cross-sectional study	4	1.88 (-0.68, 4.44)	0.150	37.50%	0.187	0.304
	Cohort study	2	0.54 (0.44, 0.64)	<0.001	0.00%	0.574	
NO_2	Cross-sectional study	6	1.74 (0.09, 3.39)	0.039	93.89%	<0.001	0.463
	Cohort study	6	0.71 (-1.51, 2.92)	0.532	98.08%	<0.001	

Grouping variable	Grouping criterion	No. of studies	Pooled %-change (95% CI)	*P*-value	I^2	*P*-value for heterogeneity	*P*-value for subgroup difference
O_3	Cross-sectional study	3	5.47 (-0.54, 11.48)	0.075	92.65%	<0.001	0.435
	Cohort study	5	2.38 (-2.52, 7.28)	0.341	99.01%	<0.001	
Exposure assessment method							
$PM_{2.5}$	Fixed-site monitoring	5	4.20 (-0.14, 8.54)	0.058	95.94%	<0.001	0.210
	Model estimation	24	1.38 (0.62, 2.14)	0.001	98.35%	<0.001	
PM_{10}	Fixed-site monitoring	3	2.26 (-0.16, 4.68)	0.067	97.32%	<0.001	0.253
	Model estimation	10	0.80 (0.14, 1.46)	0.017	91.97%	<0.001	
SO_2	Fixed-site monitoring	3	0.54 (0.44, 0.64)	<0.001	0.00%	0.639	0.368
	Model estimation	3	1.74 (-0.88, 4.37)	0.193	52.64%	0.121	
NO_2	Fixed-site monitoring	4	0.67 (-2.93, 4.27)	0.715	96.58%	<0.001	0.650
	Model estimation	8	1.56 (0.25, 2.86)	0.019	97.50%	<0.001	
O_3	Fixed-site monitoring	3	2.49 (-4.53, 9.50)	0.488	97.07%	<0.001	0.708
	Model estimation	5	4.10 (-0.62, 8.82)	0.089	98.96%	<0.001	
Study quality							
PM_1	Moderate	4	1.81 (-0.07, 3.69)	0.059	86.92%	<0.001	0.110
	High	1	3.57 (2.52, 4.62)	<0.001	—	—	

Grouping variable	Grouping criterion	No. of studies	Pooled %-change (95% CI)	*P*-value	I^2	*P*-value for heterogeneity	*P*-value for subgroup difference
$PM_{2.5}$	Moderate	15	1.61 (0.38, 2.83)	0.010	93.83%	<0.001	0.852
	High	14	1.76 (0.79, 2.72)	<0.001	98.98%	<0.001	
PM_{10}	Moderate	9	1.37 (0.42, 2.32)	0.005	94.36%	<0.001	0.513
	High	4	0.79 (-0.69, 2.26)	0.297	93.48%	<0.001	
SO_2	Moderate	4	1.88 (-0.68, 4.44)	0.150	37.50%	0.187	0.304
	High	2	0.54 (0.44, 0.64)	<0.001	0.00%	0.574	
NO_2	Moderate	6	1.74 (0.09, 3.39)	0.039	93.89%	<0.001	0.463
	High	6	0.71 (-1.51, 2.92)	0.532	98.08%	<0.001	
O_3	Moderate	3	5.47 (-0.54, 11.48)	0.075	92.65%	<0.001	0.435
	High	5	2.38 (-2.52, 7.28)	0.341	99.01%	<0.001	
No. of controlled confounders							
PM_1	≥10	3	2.43 (0.53, 4.34)	0.012	92.26%	<0.001	0.926
	<10	2	2.21 (-2.19, 6.61)	0.325	86.62%	0.006	
$PM_{2.5}$	≥10	17	1.10 (0.20, 2.01)	0.016	98.78%	<0.001	0.047
	<10	12	2.97 (1.37, 4.57)	<0.001	93.06%	<0.001	
PM_{10}	≥10	5	0.25 (-0.24, 0.73)	0.316	93.46%	<0.001	0.007

Grouping variable	Grouping criterion	No. of studies	Pooled %-change (95% CI)	*P*-value	I^2	*P*-value for heterogeneity	*P*-value for subgroup difference
PM_{10}	<10	8	1.82 (0.77, 2.87)	0.001	95.41%	<0.001	0.007
SO_2	≥10	2	0.50 (0.37, 0.64)	<0.001	22.39%	0.256	0.032
	<10	4	3.67 (0.78, 6.55)	0.013	0.00%	0.982	
NO_2	≥10	5	1.58 (-0.34, 3.49)	0.106	98.63%	<0.001	0.681
	<10	7	0.98 (-1.10, 3.07)	0.356	93.61%	<0.001	
O_3	≥10	5	2.97 (-1.45, 7.39)	0.188	98.95%	<0.001	0.728
	<10	3	4.56 (-3.26, 12.38)	0.253	95.54%	<0.001	

Abbreviations: CI, confidence interval; NO_2, nitrogen dioxide; O_3, ozone; PM_1, particulate matter with an aerodynamic diameter of ≤1 µm; $PM_{2.5}$, particulate matter with an aerodynamic diameter of ≤2.5 µm; PM_{10}, particulate matter with an aerodynamic diameter of ≤10 µm; SO_2, sulfur dioxide.

Table S5. Meta-regression analysis for the association between long-term exposure to ambient air pollution and FBG.

Grouping variable	Pollutant	Grouping criterion	No. of studies	Pooled %-change (95% CI)	*P*-value [a]	I^{2} [b]
Study area	$PM_{2.5}$	Asia	22	1.32 (-0.77, 3.46)	0.434	99.93%
		Europe	2	1.91 (-2.58, 6.61)		
		America	5	Ref.		
	PM_{10}	Asia	9	1.83 (-0.90, 4.63)	0.165	99.95%
		Europe	3	3.04 (-0.13,6.31)		
		America	1	Ref.		
	SO_2	Europe	1	2.63 (-2.80, 8.37)	0.350	3.85%
		Asia	5	Ref.		
	NO_2	Europe	4	-0.81 (-4.70, 6.64)	0.767	99.76%
		Asia	7	1.70(-3.65, 7.34)		
		America	1	Ref.		
	O_3	Europe	1	-2.97 (-13.67, 9.05)	0.613	99.84%
		Asia	7	Ref.		
Sample size	PM_1	≥5000	3	0.57 (-3.26,4.55)	0.774	91.22%
		<5000	2	Ref.		
	$PM_{2.5}$	≥5000	12	-0.23 (-1.84, 1.40)	0.782	99.92%

Grouping variable	Pollutant	Grouping criterion	No. of studies	Pooled %-change (95% CI)	*P*-value [a]	I^2 [b]
		<5000	17	Ref.		
	PM_{10}	≥5000	5	0.48 (-1.11, 2.10)	0.555	99.78%
		<5000	8	Ref.		
	SO_2	≥5000	2	-1.15 (-4.58, 2.41)	0.522	33.01%
		<5000	4	Ref.		
	NO_2	≥5000	6	-0.90 (-3.72, 2.00)	0.537	99.47%
		<5000	6	Ref.		
	O_3	≥5000	5	-3.47 (-10.83, 4.50)	0.384	99.46%
		<5000	3	Ref.		
Participant type	PM_1	Pregnant women	1	-2.53 (-5.90, 0.96)	0.153	86.40%
		General population	4	Ref.		
	$PM_{2.5}$	Pregnant women	7	-0.21 (-2.02, 1.64)	0.823	99.92%
		General population	22	Ref.		
	PM_{10}	Pregnant women	2	0.09 (-2.01, 2.23)	0.935	99.95%
		General population	11	Ref.		
	SO_2	Pregnant women	1	2.30 (-5.41, 10.64)	0.570	3.36%
		General population	5	Ref.		

Grouping variable	Pollutant	Grouping criterion	No. of studies	Pooled %-change (95% CI)	*P*-value [a]	I^2 [b]
	NO_2	Pregnant women	1	-4.82 (-8.61, -0.87)	0.017	99.66%
		General population	11	Ref.		
	O_3	Pregnant women	2	-5.81 (-12.80, 1.75)	0.129	99.74%
		General population	6	Ref.		
Mean/median age	PM_1	19-64y	4	-1.31 (-5.96, 3.58)	0.594	98.58%
		≤18y	1	Ref.		
	$PM_{2.5}$	19-64y	22	-0.49 (-2.52, 1.58)	0.089	99.93%
		≥65y	2	3.88 (-0.37, 8.30)		
		≤18y	5	Ref.		
	PM_{10}	19-64y	10	-0.32 (-2.12, 1.53)	0.034	99.94%
		≥65y	2	3.24 (0.14,6.43)		
		≤18y	2	Ref.		
	SO_2	19-64y	5	-4.36 (-15.03, 7.64)	0.459	3.23%
		≥65y	1	Ref.		
	NO_2	≥65y	1	6.03 (-0.50, 12.99)	0.127	99.72%
		19-64y	10	0.93 (-3.46, 5.51)		
		≤18y	1	Ref.		

Grouping variable	Pollutant	Grouping criterion	No. of studies	Pooled %-change (95% CI)	*P*-value [a]	I^2 [b]
	O_3	19-64y	7	-6.92 (-16.74, 4.06)	0.207	99.80%
		≥65y	1	Ref.		
Female proportion	PM_1	≥50%	4	1.85 (-2.37, 6.25)	0.396	91.17%
		<50%	1	Ref.		
	$PM_{2.5}$	≥50%	22	-0.37 (-2.29, 1.58)	0.706	99.93%
		<50%	7	Ref.		
	PM_{10}	≥50%	10	-0.83 (-2.60, 0.97)	0.364	99.95%
		<50%	3	Ref.		
	SO_2	≥50%	4	1.13 (-2.38, 4.77)	0.533	32.46%
		<50%	2	Ref.		
	NO_2	≥50%	9	-1.50 (-4.63, 1.73)	0.358	99.74%
		<50%	3	Ref.		
	O_3	≥50%	6	-1.69 (-10.27, 7.72)	0.715	99.56%
		<50%	2	Ref.		
Study design	PM_1	Cross-sectional study	4	-1.74 (-5.57, 2.24)	0.385	98.15%
		Cohort study	1	Ref.		
	$PM_{2.5}$	Cross-sectional	15	0.28 (-1.32, 1.90)	0.737	99.92%

Grouping variable	Pollutant	Grouping criterion	No. of studies	Pooled %-change (95% CI)	*P*-value [a]	I^2 [b]
		Cohort study	14	Ref.		
	PM_{10}	Cross-sectional study	9	0.59 (-1.12, 2.34)	0.500	99.80%
		Cohort study	4	Ref.		
	SO_2	Cross-sectional study	4	0.97 (-2.76, 4.83)	0.615	36.34%
		Cohort study	2	Ref.		
	NO_2	Cross-sectional study	6	1.13 (-1.71, 4.06)	0.439	99.45%
		Cohort study	6	Ref.		
	O_3	Cross-sectional study	3	7.50 (1.62, 13.72)	0.012	99.11%
		Cohort study	5	Ref.		
Exposure assessment method	$PM_{2.5}$	Model estimation	24	-1.00 (-3.29, 1.35)	0.403	99.93%
		Fixed-site monitoring	5	Ref.		
	PM_{10}	Model estimation	10	-1.26 (-2.94, 0.44)	0.145	99.76%
		Fixed-site monitoring	3	Ref.		
	SO_2	Model estimation	3	0.60 (-2.96, 4.29)	0.744	34.43%
		Fixed-site monitoring	3	Ref.		
	NO_2	Model estimation	8	1.02 (-1.94, 4.08)	0.502	99.45%
		Fixed-site monitoring	4	Ref.		

Grouping variable	Pollutant	Grouping criterion	No. of studies	Pooled %-change (95% CI)	*P*-value [a]	I^2 [b]
	O_3	Model estimation	5	1.69 (-6.20, 10.25)	0.684	99.51%
		Fixed-site monitoring	3	Ref.		
Study quality	PM_1	Moderate	4	-1.74 (-5.57, 2.24)	0.385	98.15%
		High	1	Ref.		
	$PM_{2.5}$	Moderate	15	0.28 (-1.32, 1.90)	0.737	99.92%
		High	14	Ref.		
	PM_{10}	Moderate	9	0.59 (-1.12, 2.34)	0.500	99.80%
		High	4	Ref.		
	SO_2	Moderate	4	0.97 (-2.76, 4.83)	0.615	36.34%
		High	2	Ref.		
	NO_2	Moderate	6	1.13 (-1.71, 4.06)	0.439	99.45%
		High	6	Ref.		
	O_3	Moderate	3	7.50 (1.62, 13.72)	0.012	99.11%
		High	5	Ref.		
No. of controlled confounders	PM_1	≥10	3	0.57 (-3.26, 4.55)	0.774	91.22%
		<10	2	Ref.		
	$PM_{2.5}$	≥10	17	-1.57 (-3.13, 0.01)	0.051	99.92%

Grouping variable	Pollutant	Grouping criterion	No. of studies	Pooled %-change (95% CI)	*P*-value [a]	I^2 [b]
		<10	12	Ref.		
	PM_{10}	≥10	5	-1.48 (-2.80, -0.13)	0.031	99.92%
		<10	8	Ref.		
	SO_2	≥10	2	-3.12 (-5.87, -0.28)	0.032	6.68%
		<10	4	Ref.		
	NO_2	≥10	5	0.63 (-2.24, 3.57)	0.671	99.73%
		<10	7	Ref.		
	O_3	≥10	5	-1.47 (-9.30, 7.04)	0.726	99.84%
		<10	3	Ref.		

[a] Test for subgroup difference.

[b] Estimate for how much of the unaccounted variability (residual heterogeneity + sampling error) is attributable to residual heterogeneity.

Abbreviations: CI, confidence interval; NO_2, nitrogen dioxide; O_3, ozone; PM_1, particulate matter with an aerodynamic diameter of ≤1 µm; $PM_{2.5}$, particulate matter with an aerodynamic diameter of ≤2.5 µm; PM_{10}, particulate matter with an aerodynamic diameter of ≤10 µm; SO_2, sulfur dioxide.

Table S6. Publication bias of the included studies.

Pollutant	No. of studies	Begg's test *P*-value	Egger's test *P*-value
PM_1	5	—	—
$PM_{2.5}$	29	0.574	0.674
PM_{10}	13	0.903	0.008
SO_2	6	—	—
NO_2	12	1.000	0.112
O_3	8	—	—

Note: Begg's test and Egger's test were unable to be conducted for several associations due to the small numbers of studies included.

Abbreviations: NO_2, nitrogen dioxide; O_3, ozone; PM_1, particulate matter with an aerodynamic diameter of ≤1 μm; $PM_{2.5}$, particulate matter with an aerodynamic diameter of ≤2.5 μm; PM_{10}, particulate matter with an aerodynamic diameter of ≤10 μm; SO_2, sulfur dioxide.

Table S7. Results of sensitivity analyses omitting one study each at a time.

Pollutant	Study	Pooled %-change (95% CI)	*P*-value	I^2
PM_1	Omitting Liu F et al. (2019)	1.83 (-0.07, 3.76)	0.059	86.90%
PM_1	Omitting Mei et al. (2023)	1.84 (0.13, 3.57)	0.035	94.30%
PM_1	Omitting Najafi et al. (2020)	2.85 (1.04, 4.68)	0.002	90.20%
PM_1	Omitting Yang et al. (2018)	2.72 (0.63, 4.87)	0.011	94.10%
PM_1	Omitting Zhang et al. (2019)	2.05 (0.07, 4.08)	0.042	94.40%
PM_1	Pooled estimate	2.24 (0.54, 3.96)	0.010	93.30%
—	—	—	—	—
$PM_{2.5}$	Omitting Alderete et al. (2017)	1.80 (0.97, 2.63)	<0.001	98.20%
$PM_{2.5}$	Omitting Cai L et al. (2019)	1.71 (0.88, 2.55)	<0.001	98.20%
$PM_{2.5}$	Omitting Chen et al (2024)	1.75 (0.92, 2.59)	<0.001	98.20%
$PM_{2.5}$	Omitting Chuang et al. (2011)	1.56 (0.82, 2.30)	<0.001	98.20%
$PM_{2.5}$	Omitting Curto et al. (2019)	1.71 (0.92, 2.51)	<0.001	98.20%
$PM_{2.5}$	Omitting Du et al. (2021)	1.72 (0.89, 2.56)	<0.001	98.20%
$PM_{2.5}$	Omitting Erqou et al. (2018)	1.66 (0.88, 2.45)	<0.001	98.20%
$PM_{2.5}$	Omitting Feizi et al. (2023)	1.79 (0.96, 2.62)	<0.001	98.20%
$PM_{2.5}$	Omitting Holliday et al. (2019)	1.84 (1.05, 2.63)	<0.001	98.20%
$PM_{2.5}$	Omitting Kang et al. (2020)	1.79 (0.96, 2.63)	<0.001	98.20%
$PM_{2.5}$	Omitting Li et al. (2018)	1.82 (1.01, 2.63)	<0.001	98.20%
$PM_{2.5}$	Omitting Lin et al. (2020)	1.62 (0.83, 2.43)	<0.001	98.20%
$PM_{2.5}$	Omitting Liu C et al. (2016)	1.77 (0.94, 2.61)	<0.001	98.20%
$PM_{2.5}$	Omitting Liu F et al. (2019)	1.41 (0.76, 2.07)	<0.001	98.00%
$PM_{2.5}$	Omitting Liu F et al. (2022)	1.81 (0.99, 2.63)	<0.001	93.70%
$PM_{2.5}$	Omitting Liu R et al. (2022)	1.77 (0.97, 2.57)	<0.001	98.20%
$PM_{2.5}$	Omitting Lu et al. (2017)	1.56 (0.80, 2.33)	<0.001	98.10%
$PM_{2.5}$	Omitting Mei et al. (2023)	1.78 (0.97,2.61)	<0.001	98.20%
$PM_{2.5}$	Omitting Najafi et al. (2020)	1.80 (0.98, 2.63)	<0.001	98.10%
$PM_{2.5}$	Omitting Pablo et al (2024)	1.70 (0.88, 2.53)	<0.001	98.20%
$PM_{2.5}$	Omitting Shen et al. (2024)	1.79 (0.96, 2.62)	<0.001	95.60%
$PM_{2.5}$	Omitting Wang et al. (2020)	1.77 (0.94, 2.61)	<0.001	98.20%
$PM_{2.5}$	Omitting Wolf et al. (2016)	1.67 (0.88, 2.46)	<0.001	98.20%
$PM_{2.5}$	Omitting Yang et al. (2018)	1.79 (0.96, 2.62)	<0.001	98.20%
$PM_{2.5}$	Omitting Ye et al. (2020)	1.80 (0.98, 2.63)	<0.001	98.20%
$PM_{2.5}$	Omitting Yu et al. (2020)	1.66 (0.86, 2.46)	<0.001	98.20%

Pollutant	Study	Pooled %-change (95% CI)	*P*-value	I^2
$PM_{2.5}$	Omitting Zhang et al. (2019)	1.68 (0.87, 2.50)	<0.001	98.20%
$PM_{2.5}$	Omitting Zhang S et al. (2021)	1.76 (0.94, 2.58)	<0.001	98.20%
$PM_{2.5}$	Omitting Zhang Y et al. (2020)	1.69 (0.87, 2.51)	<0.001	98.20%
$PM_{2.5}$	Pooled estimate	1.72 (0.93, 2.52)	<0.001	98.10%
—	—	—	—	—
PM_{10}	Omitting Cai L et al. (2019)	1.72 (0.93, 2.52)	<0.001	98.10%
PM_{10}	Omitting Cai Y et al. (2017)	1.23 (0.37, 2.10)	0.005	94.60%
PM_{10}	Omitting Chuang et al. (2011)	1.06 (0.26, 1.86)	0.009	93.00%
PM_{10}	Omitting Feizi et al. (2023)	0.91 (0.26, 1.56)	0.006	94.00%
PM_{10}	Omitting Holliday et al. (2019)	1.31 (0.48, 2.15)	0.002	94.20%
PM_{10}	Omitting Lin et al. (2020)	1.34 (0.56, 2.13)	0.001	94.90%
PM_{10}	Omitting Liu F et al. (2022)	1.05 (0.26, 1.85)	0.009	93.60%
PM_{10}	Omitting Mei et al. (2023)	1.31 (0.49, 2.15)	0.002	93.80%
PM_{10}	Omitting Najafi et al. (2020)	1.24 (0.40, 2.08)	0.004	94.90%
PM_{10}	Omitting Riant et al. (2018)	1.31 (0.48, 2.15)	0.002	94.90%
PM_{10}	Omitting Wolf et al. (2016)	1.11 (0.33, 1.90)	0.005	94.80%
PM_{10}	Omitting Yang et al. (2018)	1.19 (0.37, 2.03)	0.005	94.90%
PM_{10}	Omitting Zhang et al. (2019)	1.24 (0.39, 2.11)	0.004	94.30%
PM_{10}	Pooled estimate	1.19 (0.41, 1.97)	0.003	94.40%
—	—	—	—	—
SO_2	Omitting Chuang et al. (2011)	0.51 (0.40, 0.63)	<0.001	27.20%
SO_2	Omitting Feizi et al. (2023)	1.90 (-0.47, 4.33)	0.117	22.40%
SO_2	Omitting Lin et al. (2020)	0.51 (0.40, 0.63)	<0.001	30.10%
SO_2	Omitting Mei et al. (2023)	0.51 (0.38, 0.63)	<0.001	0.00%
SO_2	Omitting Riant et al. (2018)	0.51 (0.39, 0.63)	<0.001	22.60%
SO_2	Omitting Yang et al. (2018)	1.90 (-0.34, 4.20)	0.098	14.80%
SO_2	Pooled estimate	0.52 (0.40, 0.63)	<0.001	17.20%
—	—	—	—	—
NO_2	Omitting Alderete et al. (2017)	1.37 (-0.15, 2.91)	0.078	97.40%
NO_2	Omitting Cai Y et al. (2017)	1.30 (-0.23, 2.86)	0.097	97.30%
NO_2	Omitting Chuang et al. (2011)	0.85 (-0.42, 2.14)	0.191	97.30%
NO_2	Omitting Feizi et al. (2023)	1.32 (-0.21, 2.88)	0.092	97.10%
NO_2	Omitting Lin et al. (2020)	1.64 (0.42, 2.87)	0.008	97.10%
NO_2	Omitting Liu F et al. (2019)	0.78 (-0.43, 2.01)	0.207	94.30%

Pollutant	Study	Pooled %-change (95% CI)	*P*-value	I^2
NO_2	Omitting Liu F et al. (2022)	1.37 (-0.16, 2.91)	0.079	96.70%
NO_2	Omitting Mei et al. (2023)	1.17 (-0.27, 2.63)	0.111	97.40%
NO_2	Omitting Riant et al. (2018)	1.27 (-0.26, 2.82)	0.103	97.40%
NO_2	Omitting Wolf et al. (2016)	1.23 (-0.30, 2.78)	0.115	97.40%
NO_2	Omitting Yang et al. (2018)	1.19 (-0.34, 2.74)	0.128	97.30%
NO_2	Omitting Zhang S et al. (2021)	1.34 (-0.19, 2.89)	0.086	97.40%
NO_2	Pooled estimate	1.24 (-0.15, 2.65)	0.082	97.20%
—	—	—	—	—
O_3	Omitting Chuang et al. (2011)	2.67 (-1.11, 6.59)	0.169	98.60%
O_3	Omitting Feizi et al. (2023)	3.93 (-0.35, 8.40)	0.072	98.70%
O_3	Omitting Lin et al. (2020)	4.46 (0.59, 8.47)	0.024	98.50%
O_3	Omitting Liu X et al. (2022)	1.97 (-0.93, 4.97)	0.185	93.30%
O_3	Omitting Mei et al. (2023)	3.07 (-0.98, 7.29)	0.139	98.70%
O_3	Omitting Yang et al. (2018)	4.03 (-0.20, 8.45)	0.062	98.60%
O_3	Omitting Zhang L et al. (2023)	3.98 (-0.28, 8.42)	0.067	98.70%
O_3	Omitting Zhang S et al. (2021)	3.95 (-0.32, 8.39)	0.070	98.70%
O_3	Pooled estimate	3.52 (-0.22, 7.40)	0.065	98.50%

Abbreviations: CI, confidence interval; NO_2, nitrogen dioxide; O_3, ozone; PM_1, particulate matter with an aerodynamic diameter of ≤1 μm; $PM_{2.5}$, particulate matter with an aerodynamic diameter of ≤2.5 μm; PM_{10}, particulate matter with an aerodynamic diameter of ≤10 μm; SO_2, sulfur dioxide.

Table S8. Results of sensitivity analyses for different exposure windows.

Pollutant	No. of studies	Exposure metric	Percent change (95%CI)	*P*-value	I^2
$PM_{2.5}$	7	6-m average	2.37 (1.05, 3.71)	<0.001	93.20%
	18	1-y average	1.62 (0.47, 2.79)	0.006	92.70%
	4	3-y average	2.57 (-0.17, 5.39)	0.067	97.60%
	29	Main result	1.72 (0.93, 2.52)	<0.001	98.10%
PM_{10}	9	1-y average	1.32 (0.20, 2.44)	<0.001	92.20%
	13	Main result	1.19 (0.41, 1.97)	0.003	94.40%
SO_2	4	1-y average	1.94 (-0.59, 4.54)	0.135	32.10%
	6	Main result	0.52 (0.40, 0.63)	<0.001	0.172
NO_2	8	1-y average	1.18 (0.08, 2.29)	0.035	83.90%
	12	Main result	1.24 (-0.15, 2.65)	0.082	0.972
O_3	4	1-y average	4.38 (-0.18, 9.15)	0.060	87.10%
	8	Main result	3.52 (-0.22, 7.40)	0.065	0.985

Abbreviations: CI, confidence interval; NO_2, nitrogen dioxide; O_3, ozone; PM_1, particulate matter with an aerodynamic diameter of ≤1 μm; $PM_{2.5}$, particulate matter with an aerodynamic diameter of ≤2.5 μm; PM_{10}, particulate matter with an aerodynamic diameter of ≤10 μm; SO_2, sulfur dioxide.

Table S9. Results of sensitivity analyses replacing studies with the same population.

Pollutant	No. of studies	Model	Percent change (95% CI)	*P*-value	I^2
$PM_{2.5}$	29	Main result	1.72 (0.93, 2.52)	<0.001	98.10%
$PM_{2.5}$	29	Replaced by Kang et al.(2023)	1.71 (0.96, 2.47)	<0.001	98.10%

Abbreviations: CI, confidence interval; $PM_{2.5}$, particulate matter with an aerodynamic diameter of ≤2.5 µm.

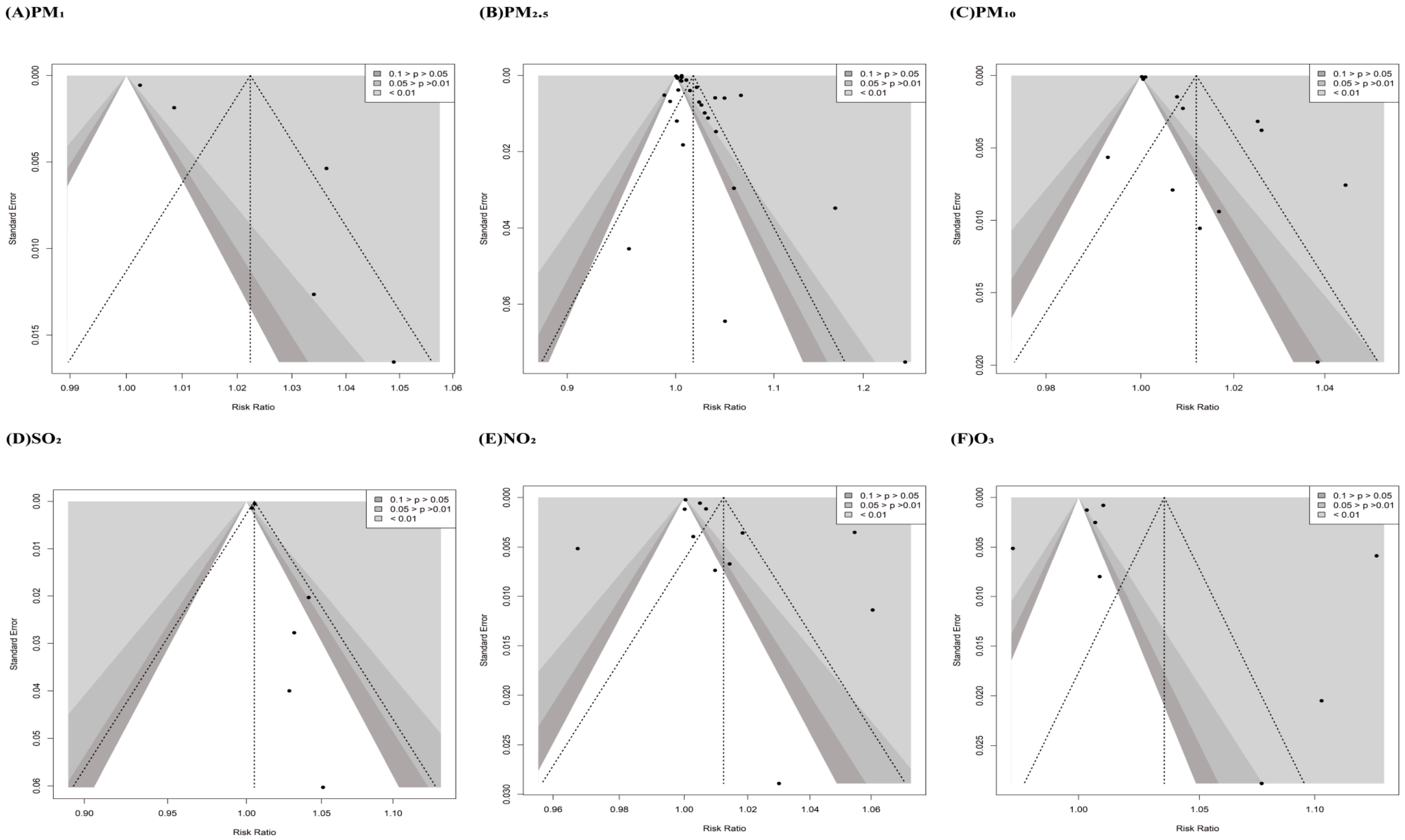

Fig. S1. Funnel plots of publication bias for the association between long-term exposure to ambient air pollution and FBG.

Abbreviations: FBG, fasting blood glucose; NO_2, nitrogen dioxide; O_3, ozone; PM_1, particulate matter with an aerodynamic diameter of ≤1 μm; $PM_{2.5}$, particulate matter with an aerodynamic diameter of ≤2.5 μm; PM_{10}, particulate matter with an aerodynamic diameter of ≤10 μm; SO_2, sulfur dioxide.
